# Upper extremity function and disability recovery with vibration therapy after stroke: a systematic review and meta-analysis of RCTs

**DOI:** 10.1186/s12984-024-01515-6

**Published:** 2024-12-21

**Authors:** Yueh-Hsun Lu, Hung-Ju Chen, Chun-De Liao, Po-Jung Chen, Xin-Miao Wang, Chieh-Hsiang Yu, Po-Yin Chen, Chueh-Ho Lin

**Affiliations:** 1 Department of Radiology, Shuang-Ho Hospital, Taipei Medical University, New Taipei City, Taiwan (R.O.C.); 2 Department of Radiology, School of Medicine, College of Medicine, Taipei Medical University, Taipei, Taiwan (R.O.C.); 3 National Center for Geriatrics and Welfare Research, National Health Research Institutes, Zhunan Town, Miaoli County, Taiwan (R.O.C.); 4 Department of Physical Medicine and Rehabilitation, Shuang Ho Hospital, Taipei Medical University, New Taipei City, Taiwan (R.O.C.); 5 Master Program in Long-Term Care, College of Nursing, Taipei Medical University, Taipei, Taiwan (R.O.C.); 6 National Center for Geriatrics and Welfare Research, National Health Research Institutes, Yunlin County, Taiwan (R.O.C.); 7 School of Health Management, College of Health Management, Shanghai Jianqiao University, Shanghai, People’s Republic of China; 8 Department of Physical Therapy and Assistive Technology, National Yang Ming Chiao Tung University, Taipei, Taiwan (R.O.C.); 9 National Center for Geriatrics and Welfare Research, National Health Research Institutes, No. 8, Xuefu W. Rd., Huwei Township, Yunlin County, 63247 Taiwan (R.O.C.); 10 Research Center in Nursing Clinical Practice, Wan Fang Hospital, Taipei Medical University, Taipei, Taiwan (R.O.C.)

**Keywords:** Stroke, Upper extremity, Quality of life, Vibration therapy, Motor function, Disability

## Abstract

**Background:**

This study aimed to investigate the therapeutic effects of vibration therapy for improving upper extremity motor impairment, function, and disability recovery in people with stroke.

**Design:**

We followed the Preferred Reporting Items for Systematic Reviews and Meta-Analysis guidelines. PubMed, EMBASE, the Cochrane Library Database, Physiotherapy Evidence Database (PEDro), China Knowledge Resource Integrated Database, and Google Scholar were searched from inception to May 31, 2024. Randomized controlled trials (RCTs) that evaluated the effects of vibration therapy on upper extremity motor impairment, function, and disability recovery post-stroke were analyzed.

**Setting and participants:**

Participants with a diagnosis of stroke with hemiplegia (or hemiparesis) were recruited.

**Methods:**

Methodological quality assessment was performed using the PEDro quality score. Upper extremity motor impairment, function, and disability were the primary outcomes. Upper extremity motor impairment was measured using the Fugl-Meyer Assessment scale and other methods. Upper extremity functions were evaluated using the Wolf Motor Function test or other tools assessing manipulative activities. Disability was assessed using the Functional Independence Measure, Barthel index, and other methods.

**Results:**

Overall, 30 RCTs including 1621 people with stroke were selected. Compared with the control, vibration therapy exerted significant effects on upper extremity motor impairment [standardized mean difference (SMD) = 1.19; *p* < 0.00001)], function (SMD = 0.62; *p* < 0.00001), and disability recovery (SMD = 1.01; *p* < 0.00001). The subgroup analysis revealed that focal vibration therapy (SMD = 2.14) had favorable effects on disability recovery compared with whole-body vibration therapy (SMD = 2.0). Interventions lasting 4–8 weeks showed significant improvements in motor impairment (SMD = 1.19), motor function (SMD = 0.57), and disability (SMD = 0.84); additionally, the effects of vibration therapy combined with conventional rehabilitation (SMD = 1.03) were superior to those of vibration therapy alone (SMD = 0.21).

**Conclusions:**

Vibration therapy may be a reliable rehabilitation program to improve upper extremity motor functions and disabilities. Furthermore, vibration therapy should be performed at the earliest possibility after stroke for at least 4–8 weeks.

*Trial registration* The protocol of this study was registered with PROSPERO (Registration number: CRD42022301119).

**Supplementary Information:**

The online version contains supplementary material available at 10.1186/s12984-024-01515-6.

## Background

Brief Summary Vibration therapy (VT) significantly reduces upper extremity (UE) motor impairment, enhances UE motor function, and improves disability outcomes in people with stroke. Combining VT with standard rehabilitation is recommended, starting as early as possible after a stroke and continuing for at least 8 weeks. Both low and high vibration frequencies are effective, but focal muscle vibration is particularly beneficial for disability recovery.

Feeding, dressing, and writing are the most common activities of daily living that require motor function and participation of the upper limbs [1, 2]. These activities are essential for maintaining independence and quality of life. Neurologic disorders such as stroke, which often results in hemiparesis, where one side of the body becomes weak or paralyzed, may lead to poor motor function, muscle weakness, spasticity in paretic limbs, and disability [1, 2].

Full recovery of motor function and disabilities of the upper limbs occurs in less than 20% of people with stroke undergoing rehabilitation programs [1, 3]. Hence, many of these people with stroke have poor motor function and disability in the upper limbs, affecting their quality of life substantially [4, 5]. This impairment often necessitates long-term rehabilitation and support to manage daily activities [2, 3]. Consequently, independence and social participation can be significantly compromised for these individuals.

Compared with previous rehabilitation programs, vibration therapy (VT) stimulates muscle activity through the excitation of the tonic vibration reflex which activates efferent Ia, resulting in α-motor neuron excitation to generate muscle fiber strength and induce motor function performance [6, 7]. This implies that weak muscle activity and motor function in paretic limbs could be improved using VT. Recent studies investigated the effects of the whole-body vibration (WBV) on improving disabilities in the upper limbs for people with stroke by asking them to sit on a chair and place their hands on the WBV platform [8, 9]. After 4 weeks of the intervention, the people with stroke in the experimental group treated with WBV showed better motor function improvements in the upper limbs than those in the control group undergoing a traditional rehabilitation program [8, 9]. Furthermore, several studies have developed focal muscle vibration (FMV) to improve motor function and disabilities in people with stroke [10–12].

However, vibration force transmission from WBV or FMV to the targeted upper limbs is different being a complex process that could be influenced by various biomechanical mechanisms and result in different outcomes according to the type of vibration application [13, 14]. Previous studies often used vibrations < 20 Hz for muscle relaxation and reduction of spasticity [15, 16]. Research also shows that vibrations in the range of 20–30 Hz can improve gait balance [17]. Since the resonance frequencies of some important human organs are between 5 and 20 Hz, previous studies have considered 20 Hz as a safety threshold for vibration frequency [18].

Hence, optimal vibration protocols to improve motor function and disabilities should be established with strong evidence before applying them to improve motor and functional recovery in individuals with stroke in the clinical setting [7, 19, 20]. Therefore, it is crucial to develop an optimal evidence-based VT protocol for improving motor function and disability recovery to help clinical therapists enhance upper limb recovery in people with stroke.

Despite the potential of VT, its use in the upper extremities (UEs) and benefits on function and disability recovery have rarely been discussed; furthermore, evidence-based treatment effects are not well-established. Therefore, this meta-analysis aimed to investigate the effects of VT protocols on UE motor impairment, function, and disability recovery in people with stroke.

## Methods

### Design

The present study followed the Preferred Reporting Items for Systematic Reviews and Meta-Analysis guidelines [21]. The protocol of this study was registered with PROSPERO (Registration number: CRD42022301119) and the need for written informed consent was waived because of its retrospective design.

Articles were screened and obtained through a comprehensive electronic search of online databases, including PubMed, EMBASE, the Cochrane Library, the Physiotherapy Evidence Database (PEDro), the China Knowledge Resource Integrated Database, and Google Scholar, from their inception to May 31, 2024. Secondary sources included papers in related systemic reviews. No restriction regarding language or time of publication was applied. Two authors (HJC and CDL) independently searched for relevant articles, screened them, and extracted data. Any disagreement between the authors was resolved through discussion. If a consensus was not reached, another team member (CHY), who served as an arbitrator, was consulted.

### Search strategy

Our key search terms were “stroke” OR “cerebral vascular accident” OR “hemiplegia” OR “hemiparesis” AND “vibration” AND “upper extremity.” The search formulas for each database are detailed in Supplementary Table 1.

### Selection criteria of the studies

Studies were eligible if they met the following criteria: randomized controlled trials (RCTs) that explored the effectiveness of VT for UEs; had adult participants with a diagnosis of ischemic or hemorrhagic stroke; the experimental group underwent VT with or without post-stroke standard rehabilitation (SR) (SR referred to conventional physiotherapy or occupational therapy, which increased UE strength, improved joint flexibility, and enhanced function); the control group received placebo vibratory stimuli, SR, or a combination of both; and those that performed a validated measurement of changes in UE motor impairment, function, or disability before and after the interventions. The definitions of these outcomes are provided in the g subsections below.

The exclusion criteria were studies that used an animal model, case reports or case series, and studies that were prospectively designed trials without a comparison group.

### Outcome measures

UE motor impairment, function, and disability were the primary outcomes in the present study. UE motor impairment was measured using the Fugl-Meyer Assessment scale [22, 23] or other tools assessing UE motor impairment. UE functions were evaluated using the Wolf Motor Function test [24] or other tools assessing manipulative activities. Disability was assessed using the Functional Independence Measure [25], Barthel index [26], and other tools.

### Data extraction

The following data were extracted from each included trial (Table 1): characteristics of the study sample and design, including the group design, the age, sex, diagnosis, stroke type, and disease onset duration in individuals with stroke; characteristics of the interventions (i.e., type of VT, treatment duration, and number of sessions); measurement time points; and main outcome measures. The follow-up duration was categorized into four types: immediate (< 1 month), short-term (≥ 1 month, < 3 months), medium-term (≥ 3 months, < 6 months), and long-term (≥ 6 months) [27]. When multiple time points were reported within the same timeframe, the longest period was selected for analysis. One author (HJC) extracted the relevant data from the included trials, and another author (CDL) reviewed the extracted data. Any disagreement between the two authors was resolved through discussion and a third author (CHY) was consulted if a consensus could not be reached. If a trial had more than one therapeutic or control intervention, each comparison was considered to be independent in the meta-analyses [28].

**Table 1:** Summary of the characteristics of the included studies

Study, first author date	Country (area)	Groups	Age (y)*	Sex (F/M)	N	Type of stroke	Dx duration (month)	Brunnstrom stage	Exercise intervention		Measured time point (week)	Primary outcome results
									Type	Frequency × duration		
Ahn	Korea	EG: VT	58.7 ± 7.1	18/12	30	IS, HS	3.4 ± 1.1	2.53 ± 0.52	WBV	5 d/wk × 4 wk	Baseline	MFT
2019	(Asia)	CG: SR	60.7 ± 5.9	17/16	30		2.8 ± 1.1	2.40 ± 0.51		(20 sessions)	Posttest: 4	
Alp	Turkey	EG: VT + SR	61.2 ± 11.0	0/10	10	IS, HS	18 (12–48)	≥ 3	WBV	3 d/wk × 4 wk	Baseline	FIM
2018	(Asia)	CG: PLA + SR	62.9 ± 8.2	2/9	11		24 (6–60)			(12 sessions)	Mid-test: 1	
											Posttest: 4, 12, 24	
Annino	Italy	EG: VT + SR	67.8 ± 8.3	5/14	19	IS, HS	33.6 ± 3.7	NR	FMV	3 d/wk × 8 wk	Baseline	BI, MT
2019	(Europe)	CG: SR	69.4 ± 10.4	3/15	18		33.5 ± 3.7			(24 sessions)	Posttest: 8	
Calabrò	America	EG: VT + SR	66.0 ± 5.0	5/5	10	IS	5 ± 2	NR	FMV	5 d/wk × 8 wk	Baseline	FMA
2017		CG: PLA + SR	67.0 ± 4.0	6/4	10		6 ± 2			(40 sessions)	Posttest: 8	FIM
											Follow-up: 12	
Caliandro	Italy	EG: VT + SR	57.4 ± 12.8	8/20	28	IS, HS	100.7 ± 82.8	NR	FMV	3 d/wk × 1 wk	Baseline	WFM
2012	(Europe)	CG: PLA + SR	61.9 ± 15.7	7/14	21		96.4 ± 66.8			(3 sessions)	Posttest: 1	
											Follow-up: 5	
Casale	Italy	EG: VT + SR	64.7 ± 5.4	6/9	15	IS, HS	Chronic	NR	FMV	5 d/wk × 2 wk	Baseline	MFT
2014	(Europe)	CG: PLA + SR	65.1 ± 5.8	6/9	15		(≥ 6)			(10 sessions)	Posttest: 2	
											Follow-up: 4	
Celletti	Italy	EG 1: VT + SMS	43 (31–68)	2/4	6	IS, HS	72 (24–396)	NR	FMV	3 d/wk × 1 wk	Baseline	WFM
2017	(Europe)	EG 2: VT + SR	43 (30–57)	2/4	6		30 (30–48)			(3 sessions)	Posttest: 1	MFT
		CG: SR	62.5 (46–69)	2/4	6		66 (46–84)					
Cordo	USA	EG: VT + SMS	56.3 ± 12.7	15/29	44	IS, HS	11.8 ± 5.7	NR	FMV	2–3 d/wk × 6–10 wk	Baseline	FMA
2022	(America)	CG: PLA + SMS	57.7 ± 12.9	16/23	39		11.3 ± 5.0			(18 sessions)	Posttest: 10	MFT
Da-Silva	UK	EG: VT	73 (65–80)	8/6	14	IS	0.9 (0.4–1.6)	NR	FMV	7 d/wk × 4 wk	Baseline	MFT, BI
2019	(Europe)	CG: PLA	69 (61–80)	12/7	19		0.9 (0.6–1.1)			(28 sessions)	Posttest: 4	
											Follow-up: 8	
Feng	China	EG: VT + VR	60.2 ± 2.9	20/31	51	IS, HS	Acute	NR	FMV	5 d/wk × 4 wk	Baseline	FMA, BI
2019	(Asia)	CG: VR	59.8 ± 4.3	19/32	51					(20 sessions)	Posttest: 4	
Hsu	Taiwan	EG: VT + SMS	53.6 ± 12.4	NR	10	IS, HS	28.9 ± 23.5	NR	FMV	2 d/wk × 6 wk	Baseline	FMA
2021	(Asia)	CG: SMS	61.7 ± 8.4		9		24.9 ± 15.3			(12 sessions)	Posttest: 6	MFT
											Follow up: 18	

Study, first author date	Country (area)	Groups	Age (y)*	Sex (F/M)	N	Type of stroke	Dx duration (month)	Brunnstrom stage	Exercise intervention		Measured time point (week)	Primary outcome results
									Type	Frequency × duration		
Lee	Korea	EG 1: VT + SR	58.5 ± 11.8	5/10	15	IS, HS	8.1 ± 4.9	NR	WBV	3 d/wk × 4 wk	Baseline	FMA, WFM
2016	(Asia)	EG 2: VT	59.2 ± 7.7	10/5	15		7.9 ± 4.2			(12 sessions)	Posttest: 4	
		CG: SR	60.2 ± 6.7	6/9	15		6.7 ± 3.9					
Li	China	EG: VT + SR	55.0 ± 10.9	2/12	14	IS, HS	1.5 ± 0.3	2-4	WBV	5 d/wk × 3 wk	Baseline	FMA
2020	(Asia)	CG: SR	56.9 ± 9.9	4/10	14		1.5 ± 0.2			(15 sessions)	Posttest: 3	
Liu	China	EG: VT + SR	57.8 ± 5.3	9/21	30	IS, HS	2.8 ± 0.3	2.86 ± 0.59	FMV	5 d/wk × 4 wk	Baseline	FMA, BI
2022	(Asia)	CG: PLA + SR	58.5 ± 4.2	10/20	30		2.7 ± 0.2	2.94 ± 0.45		(20 sessions)	Posttest: 4	
Lu	China	EG: VT + SR	55.3 ± 6.6	12/18	30	IS, HS	3.4 ± 1.2	2-3	WBV	5 d/wk × 8 wk	Baseline	FMA
2017	(Asia)	CG: SR	55.1 ± 6.3	13/17	30		3.3 ± 1.3			(40 sessions)	Posttest: 8	BI
Lu	China	EG: VT + SR	54.7 ± 4.3	16/23	39	IS, HS	0.5 ± 0.1	3-5	FMV	5 d/wk × 4 wk	Baseline	FMA, BI
2021	(Asia)	CG: SR	54.5 ± 4.3	15/24	39		0.5 ± 0.1			(20 sessions)	Posttest: 4	FIM
Meng	China	EG: VT + SR	54.7 ± 4.3	35/25	60	IS, HS	1.2 ± 0.2	NR	FMV	3 d/wk × 4 wk	Baseline	FMA, BI
2020	(Asia)	CG: SR	54.5 ± 4.3	29/31	60		1.2 ± 0.3			(12 sessions)	Posttest: 4	
Oliveira	Brazil	EG 1: VT	60.1 (55–65)	13/8	7	NR	≥ 12	≥ 4	FMV	3 d/wk × 4 wk	Baseline	WFM, MFT,
2018	(Europe)	CG 1: MrT			7					(12 sessions)	Posttest: 4	Mobility Index
		CG 2: SR			7							
Seo	American	EG: VT + SR	61.0 ± 10.0	1/5	6	IS	84 ± 84	NR	FMV	3 d/wk × 2 wk	Baseline	WFM
2019		CG: PLA + SR	64.0 ± 8.0	4/2	6		36 ± 24			(6 sessions)	Posttest: 2	MFT
											Follow up: 6	
Song	China	EG: VT + SR	77.0 ± 10.5	16/24	40	IS, HS	Subacute	1-3	FMV	5 d/wk × 3 wk	Baseline	FMA, BI
2018	(Asia)	CG: SR	75.0 ± 9.8	13/27	40					(15 sessions)	Posttest: 3	
Tavernese	Italy	EG: VT + SR	58.9 ± 14.7	3/21	24	IS, HS	Chronic	4.5 (3.0–5.3)	FMV	5 d/wk × 2 wk	Baseline	MFT
2013	(Europe)	CG: SR	58.3 ± 12.4	2/18	20		(≥ 6)	4.3 (3.0–5.0)		(10 sessions)	Posttest: 2	
Toscano	Italy	EG: VT + SR	64.7 ± 17.2	2/10	10	IS, HS	Acute	NR	FMV	1 session/d × 3 d	Baseline	FMA, MFT
2019	(Europe)	CG: PLA + SR	69.5 ± 7.3	6/6	12		(≤3 days)			(3 sessions)	Posttest: 1	
Wang	China	EG: VT + SR	48.4 ± 6.9	3/14	17	IS, HS	1.6 ± 0.3	1.6 ± 0.3	WBV	6 d/wk × 4 wk	Baseline	FMA, BI
2018	(Asia)	CG: SR	49.2 ± 7.3	3/13	16		1.6 ± 0.3	1.7 ± 0.3		(24 sessions)	Mid-test: 2	

Study, first author year	Country (area)	Groups	Age (y)*	Sex (F/M)	N	Type of stroke	Dx duration (month)	Brunnstrom stage	Exercise intervention		Measured time point (week)	Primary outcome results
									Type	Frequency × duration		
Wang	China	EG: VT + EMGB	61.3 ± 8.0	21/39	60	IS, HS	Subacute	NR	WBV	5 d/wk × 4 wk	Baseline	FMA, BI
2021	(Asia)	CG: EMGB	58.7 ± 9.3	15/45	60					(20 sessions)	Posttest: 4	
Wei	China	EG 1: VT + SR	59.2 ± 11.3	7/25	32	IS, HS	Subacute	1.6 ± 0.7	FMV	7 d/wk × 4 wk	Baseline	FMA
2019	(Asia)	CG 1: PLA + SR	60.4 ± 10.4	5/20	25			2.0 ± 1.4		(28 sessions)	Posttest: 4	FIM
		CG 2: SR	63.1 ± 10.3	4/23	27			1.8 ± 1.4			Follow up: 8, 12	
Wu	China	EG: VT + SR	58.1 ± 6.8	21/22	43	IS, HS	1.4 ± 0.1	2-4	FMV	5 d/wk × 8 wk	Baseline	FMA, BI
2016	(Asia)	CG: SR	58.4 ± 6.3	20/23	43		1.4 ± 0.1			(40 sessions)	Posttest: 8	
Wu	China	EG: VT + SR	59.3 ± 5.7	16/14	30	IS, HS	1.6 ± 0.2	NR	WBV	5 d/wk × 6 wk	Baseline	FMA, BI
2022	(Asia)	CG: SR	58.7 ± 6.4	12/18	30		1.5 ± 0.2			(30 sessions)	Posttest: 6	
Yang	China	EG 1: VT + MIT	63.2 ± 3.0	16/14	30	IS, HS	1.2 ± 0.1	NR	WBV	6 d/wk × 8 wk	Baseline	FMA, BI
2022	(Asia)	EG 2: VT	62.4 ± 3.9	12/18	30		1.2 ± 0.1			(48 sessions)	Posttest: 8	
		CG: MIT	63.8 ± 2.7	16/14	30		1.2 ± 0.2					
Yuan	China	EG: VT + SR	58.7 ± 5.4	6/17	23	IS, HS	1.6 ± 0.3	1-2	FMV	5 d/wk × 4 wk	Baseline	FMA, MFT
2018	(Asia)	CG: PLA + SR	59.2 ± 4.7	8/15	23		1.6 ± 0.3			(20 sessions)	Posttest: 4	
Zhu	China	EG 1: VT + SR	65.0 ± 5.7	8/12	20	IS, HS	3.7 ± 1.4	2-3	FMV	7 d/wk × 3 wk	Baseline	FMA
2017	(Asia)	EG 2: VT	62.0 ± 6.5	10/10	20		3.5 ± 1.5			(21 sessions)	Posttest: 3	
		CG: SR	67.0 ± 7.9	13/7	20		4.1 ± 2.3					

Values are presented as means ± standard deviations or medians (ranges or interquartile ranges)

Data present the sample mean value

F, female; M, male; Dx, disease; EG, experimental group; CG, control group; VT, vibration therapy; SR, standard rehabilitation; PLA, placebo; SMS, sensorimotor stimulation; MrT, mirror therapy; NR, not reported; IS, ischemic stroke; HS, hemorrhagic stroke; WBV, whole-body vibration; FMV, focal muscle vibration; MFT, motor function test; WFM, Wolf Motor Function test; FIM, functional independence measure; BI, Barthel index; VR, virtual reality

### Assessment of bias risks and methodological quality of the included studies

Quality assessment was performed using the PEDro quality score to assess the risk of bias. Methodological quality (MQ) of all the included studies was independently assessed by two researchers in accordance with the PEDro classification scale, which is a valid measure of the MQ of clinical trials [29]. The PEDro scale scores 10 items including: random allocation (selection bias), concealed allocation (selection bias), similarity at baseline, subject blinding (performance bias), therapist blinding (performance bias), assessor blinding (detection bias), more than 85% follow-up for at least one key outcome (attrition bias), intention-to-treat analysis (attrition bias), inter-group statistical comparison for at least one key outcome, and point and variability measures for at least one key outcome. Each item is scored as either 1 for present or 0 for absent, and a total sum score ranging 0–10 is obtained by summation of all 10 items. Based on the PEDro score, the MQ of the included RCTs was rated as high (≥ 7/10), medium (4–6/10), or low (≤ 3/10) [30].

### Data synthesis and analysis

We computed effect sizes for each study separately for the primary outcome measures (UE motor impairment, function, and disability). Primary outcome measures were defined as a pooled estimate of the mean difference in change between the mean of the treatment and the control groups. Analyses based on change scores (i.e., change from baseline) were performed to partially correct for inter-participant variability [31]. If the included studies did not present data in the manner calculated in this study, the corresponding author of the respective publication was contacted to request the research data. If no response was received, the calculations were performed using the following method: change scores were extracted whenever the mean and standard deviation (SD) of the changes were available. If the exact variance of the paired difference was not derivable, a conservative estimation was performed by assuming an intra-participant correlation coefficient of 0.7 between the baseline and post-test measured data [32]. If the SD was not reported, it was estimated by *p*-values or 95% confidence intervals (CIs). If the data was presented as medians (full ranges or interquartile ranges), they were re-calculated algebraically from the trial data to estimate the sample mean and the SD [31, 33, 34]. Owing to the diversity of measurement tools among studies for UE outcomes, all the extracted outcome data were calculated as standard mean differences (SMDs) with 95% CIs for sufficient comparability of effect sizes. We categorized the magnitude of SMDs in accordance with the following version of Cohen’s criteria [35]: trivial (*d* < 0.10), small (0.10 £ *d* < 0.25), medium (0.25 £ *d* < 0.40), and large (*d* ≥ 0.40).

Subgroup analyses were performed to identify potential factors that may affect treatment effects. These factors included the MQ level, disease stage, intervention design (i.e., monotherapy or adjunct therapy), intervention parameters, and treatment duration. The disease stage based on the time period since the onset of stroke was classified as acute (< 3 months), subacute (≥ 3 months, < 6 months), and chronic (≥ 6 months) [27]. All subgroup differences were tested for significance, and an *I*^2^ statistic was computed to estimate the degree of subgroup variability. Potential publication bias was assessed using Egger’s regression asymmetry test [36]. This meta-analysis was performed using Review Manager Software 5.4 (The Nordic Cochrane Centre, Copenhagen, Denmark).

## Results

### Trial flow

**Fig. 1:**
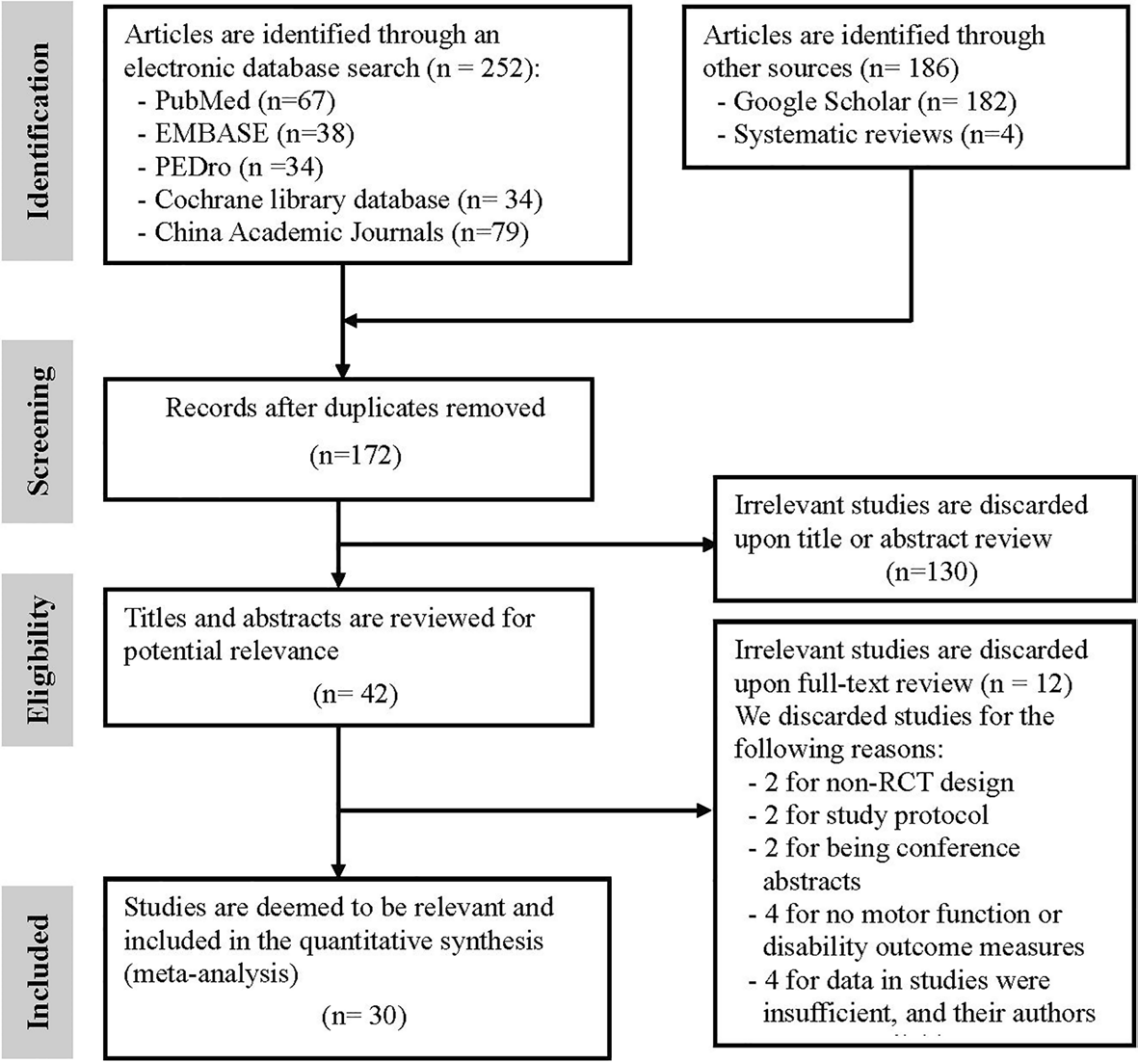
Flowchart of study selection. *PEDro* Physiotherapy Evidence Database

Figure 1 presents a flowchart of the selection process. Through the electronic and manual literature search, we identified a total of 438 articles. After removing duplicates, we reviewed the titles and abstracts of 172 studies to assess their eligibility; subsequently, 40 were considered to be relevant for full-text assessment. The final sample comprised 30 RCTs [8–12, 37–61], which were published between 2012 and 2024.

### Study characteristics

Table 1 summarizes the study characteristics and demographic data of the participants with stoke in the included RCTs. In total, 1,621 participants who had a diagnosis of stroke with hemiplegia (or hemiparesis) were recruited, with a mean age of 60.4 years (range: 43.0–77.0 years). Twelve RCTs [42–45, 47, 48, 53, 54, 57–60] enrolled people with stroke who had acute onset stroke from a few days to 3 months, whereas the other 6 RCTs [9, 46, 51, 55, 56, 61] and 12 RCTs [8, 10–12, 30, 37–41, 50, 52] enrolled people with stroke in the subacute (disease duration: < 6 months) and chronic stage (mean disease duration: 6–100 months), respectively.

Most of the included RCTs used VT as an adjunct therapy with SR [8, 10, 11, 37–40, 42–48, 50–54, 56–58, 60, 61] or other active treatments [12, 40, 41, 55, 59], whereas 6 RCTs [8, 9, 30, 42, 59, 61] used VT as monotherapy. Most of the included RCTs conducted an immediate or short-term follow-up within 3 months, four RCTs had a medium-term follow-up duration of 12–18 weeks [10, 12, 37, 56], and only one RCT had a long-term follow-up period of 6 months [37].

### Protocol of VT

A summary of the protocols for VT is presented in Table 1, and details of the protocol for VT are presented in Supplementary Table 2. Regarding the vibration mode, most of the included RCTs used FMV [10–12, 30, 38–43, 45, 47, 48, 50–53, 56, 57, 60, 61], whereas nine used WBV [8, 9, 37, 44, 46, 54, 55, 58, 59]. The most frequently used brand for FMV research was Panasonic (n = 2) [43, 57], with the model being EV2610 (n = 2) [43, 57]. The most frequently used brand for WBV research was Novotec Medical GmbH (n = 4) [8, 9, 44, 58], with the model being Galileo Med M Plus (n = 2) [44, 58].

The frequency of the vibratory wave applied ranged from 3 to 500 Hz among the included trials. Generally, seven RCTs [8, 9, 44, 45, 54, 55, 58] used low-frequency VT (≤ 20 Hz), and the other 23 RCTs [10–12, 30, 37–43, 46, 47, 50–53, 55–57, 59–61] employed a high-frequency VT (> 20 Hz). The duration of each vibration application ranged from 5 min to 3 h per training day. VT was mostly performed immediately following SR [8, 12, 37–39, 42, 43, 45–47, 51, 52, 54, 57–59], although three RCTs [10, 50, 56] used VT simultaneously with SR and two RCTs prescribed VT prior to each SR session [9, 11]. All people with stroke received VT in the seated [8–10, 12, 30, 41, 44, 45, 48, 50, 51, 54, 59, 61], supine [11, 40, 43, 47, 51–53, 57, 61], or standing position [37, 46, 55, 58, 59]. In addition, nine RCTs [11, 39, 40, 44, 50–53, 61] had a treatment duration of less than 4 weeks (3–21 sessions), whereas 13 RCTs [8, 9, 12, 30, 37, 42, 43, 45, 47, 48, 54–56, 60] and seven RCTs [10, 38, 41, 46, 57–59] employed 4-week (12–28 sessions) and 8-week (18–48 sessions) interventions of VT, respectively. Furthermore, in most of the included RCTs, the people with stroke in the control group received no treatment related to VT, whereas 11 RCTs [10, 11, 37, 39, 41, 42, 45, 50, 53, 56, 60] conducted placebo VT for the people with stroke in the control group.

### Risk of bias in the included studies

Individual PEDro scores are displayed in Supplementary Table 3. Overall, the MQ assessment revealed that 14 (46.7%) and 16 (54.3%) of the 30 included RCTs were classified as high [8, 10–12, 37, 39–41, 45, 50, 52, 53, 56, 60] and medium [9, 30, 38, 42–44, 46–48, 51, 54, 55, 57–59, 61], respectively, with a median PEDro score of 6/10 (range: 5/10–9/10). The inter-rater reliability of the cumulative PEDro scores was acceptable with an intraclass correlation coefficient of 0.88 (95% CI 0.74–0.95). All of the 30 included RCTs used random allocation, similarity at the baseline, and point estimates and variability; 6 of the 14 high-quality RCTs [11, 12, 40, 52, 56, 61] performed allocation concealment, whereas no medium-quality RCT did. Overall, the people with stroke and assessors were blinded to the study group allocations in 11 RCTs [10, 11, 37, 39, 41, 42, 45, 50, 53, 56, 60] and 13 RCTs [8, 10–12, 37–42, 50, 52, 53, 56], respectively; in addition, therapist blinding was used in 2 high-quality RCTs [50, 53], indicating potential performance bias.

### Effectiveness on UE motor impairment

**Fig. 2:**
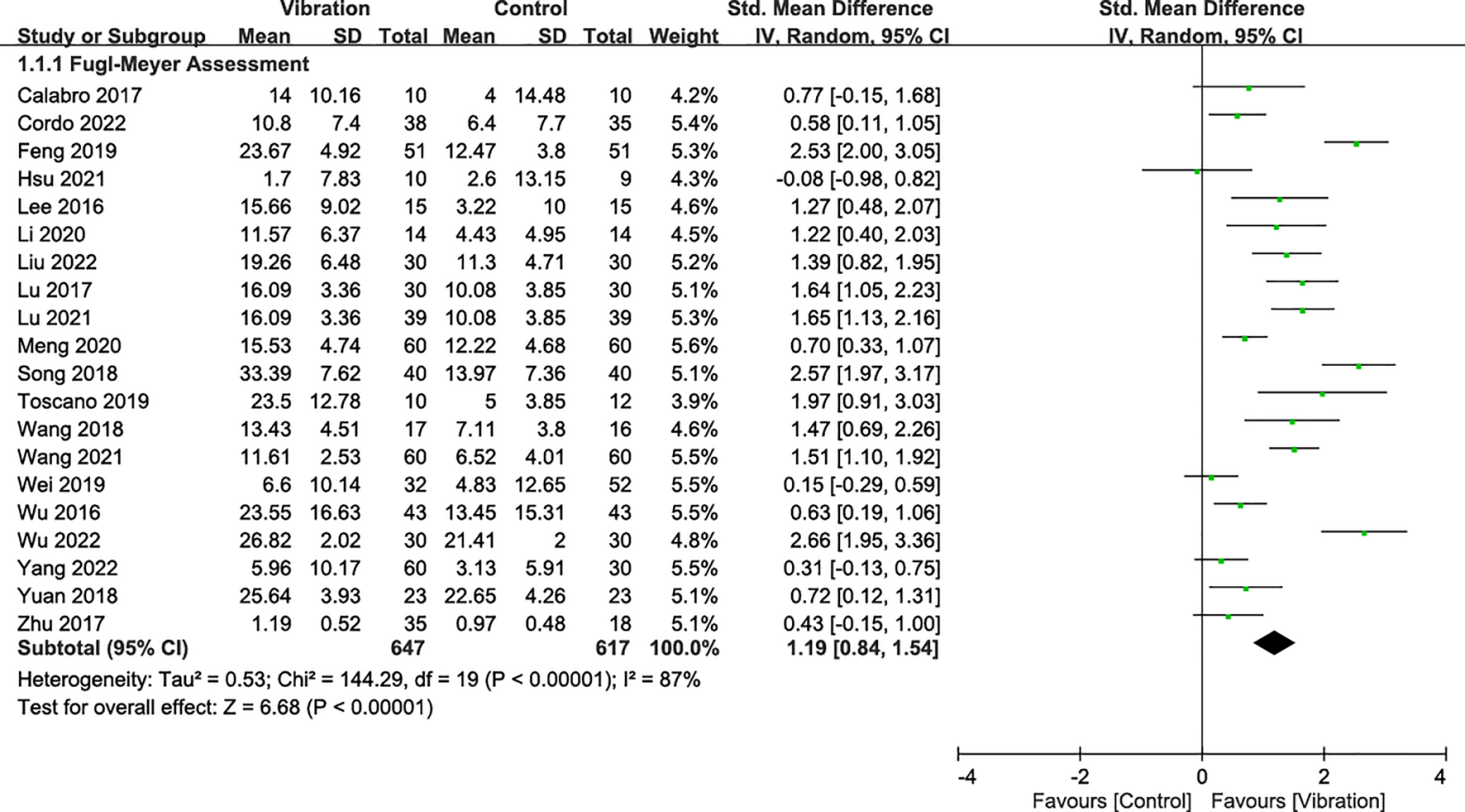
Forest plot showing the effects of vibration therapy on upper-extremity motor impairment

Upper-limb motor impairment was assessed using the Fugl-Meyer Assessment scale (in 20 RCTs [8, 10, 12, 41, 43–48, 51, 53–61]). The combined analysis showed that VT exerted significant effects on decreases in UE motor impairment with a pooled SMD of 1.19 (95% CI 0.84–1.54; *p* < 0.00001; *I*^2^ = 87%) during the overall follow-up period regardless of the type of vibration, intervention mode, and disease stage (Fig. 2).

Results of subgroup analyses for the MQ level, disease stage, and intervention factors (i.e., treatment design, vibration type, vibration frequency, treatment duration, and follow-up time) are presented in Supplementary Table 4. Significant differences in the effect of VT on UE motor impairment were observed between the subgroups based on the follow-up duration (*I*^2^: 90%; *p* < 0.00001) and treatment design (*I*^2^: 91%; *p* = 0.0009). Vibration was effective across all disease stages, including acute (SMD = 1.35), subacute (SMD = 1.25), and chronic (SMD = 0.64), with no significant differences between them. Both FMV (SMD = 1.07) and WBV (SMD = 1.39) showed significant effects, with no differences between them. Vibration frequencies ≤ 20 Hz (SMD = 1.46) and > 20 Hz (SMD = 1.05) were both effective, with no significant differences between them. However, the benefit of adjunct therapy was significant, with an SMD of 1.18. Significant effects in favor of VT were observed in the immediate (SMD = 1.51) and short-term follow-up periods (SMD = 1.12), but not in the medium-term follow-up period. Intervention durations of less than 4 weeks (SMD = 1.51), 4–8 weeks (SMD = 1.19), and 8 weeks or more (SMD = 0.89) showed significant improvements in motor impairment. Additionally, people with stroke had better outcomes (SMD = 1.32) in response to VT combined with SR than those who received VT alone (SMD = 0.39).

### Effectiveness on UE motor function

**Fig. 3:**
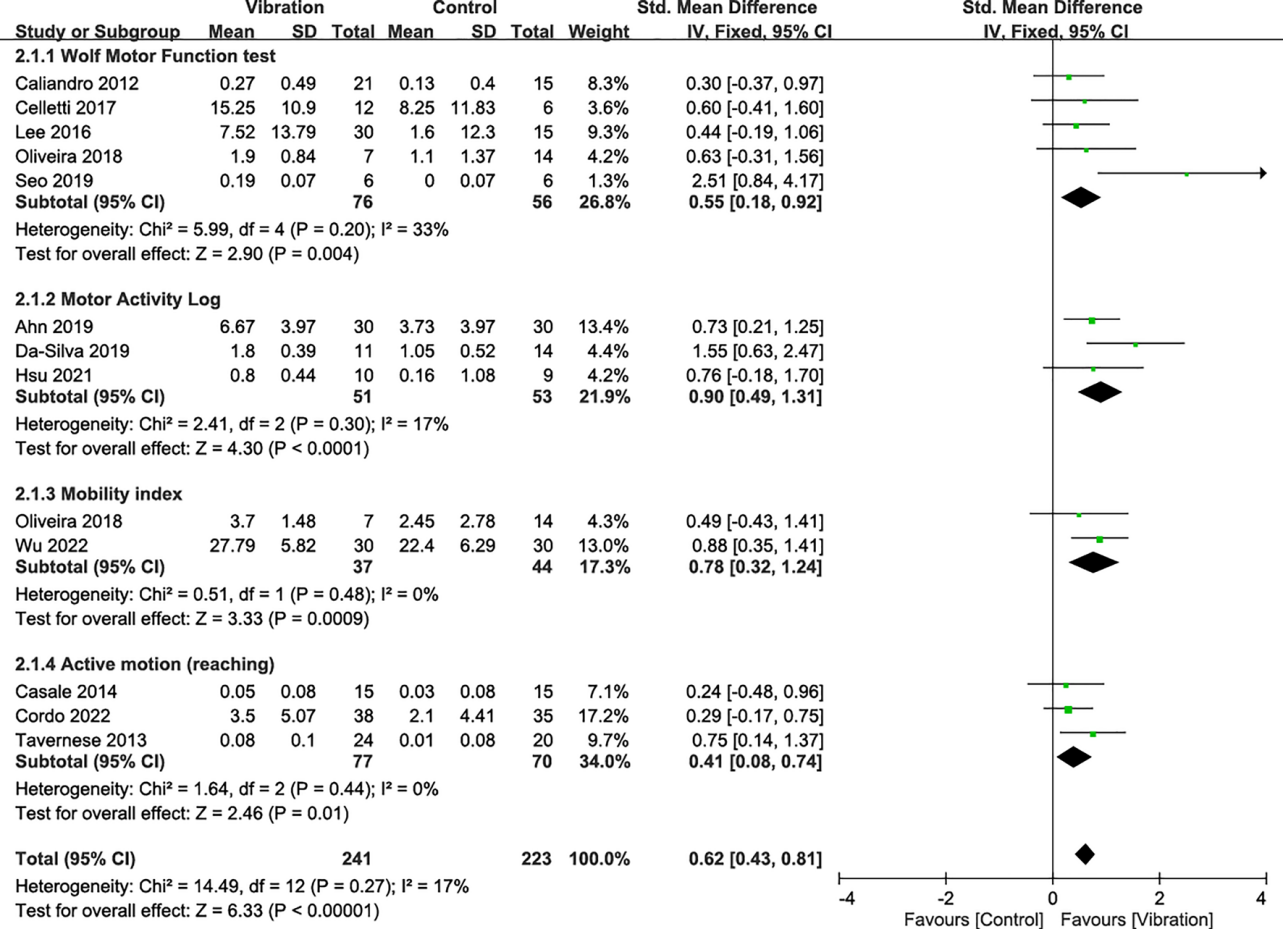
Forest plot showing the effects of vibration therapy on upper-extremity motor function

UE motor function was assessed using the Wolf Motor Function test (5 RCTs [8, 11, 30, 40, 50]), Motor Activity Log (3 RCTs [9, 12, 42]), mobility index (2 RCTs [49, 58]), and active arm motion tests (3 RCTs [39, 41, 52]). Meta-analysis results showed that VT obtained favorable effects on score changes in the Wolf Motor Function test (SMD = 0.55; *p* = 0.004), Motor Activity Log test (SMD = 0.90; *p* < 0.0001), mobility index (SMD = 0.78; *p* = 0.0009), and active arm motion tasks (SMD = 0.41; *p* = 0.01) compared with the control groups (Fig. 3). The combined analysis showed that VT exerted significant effects on increases in UE motor function with a pooled SMD of 0.62 (95% CI 0.43–0.81; *p* < 0.00001) during the overall follow-up period regardless of the type of vibration, intervention mode, and disease stage (Fig. 3). Subgroup analysis results showed no significant factor affecting treatment effects on UE motor function (Supplementary Table 4). However, only the 4–8 weeks duration (SMD = 0.57) demonstrated slightly superior outcomes in motor function (Supplementary Table 4).

### Effectiveness on disability

**Fig. 4:**
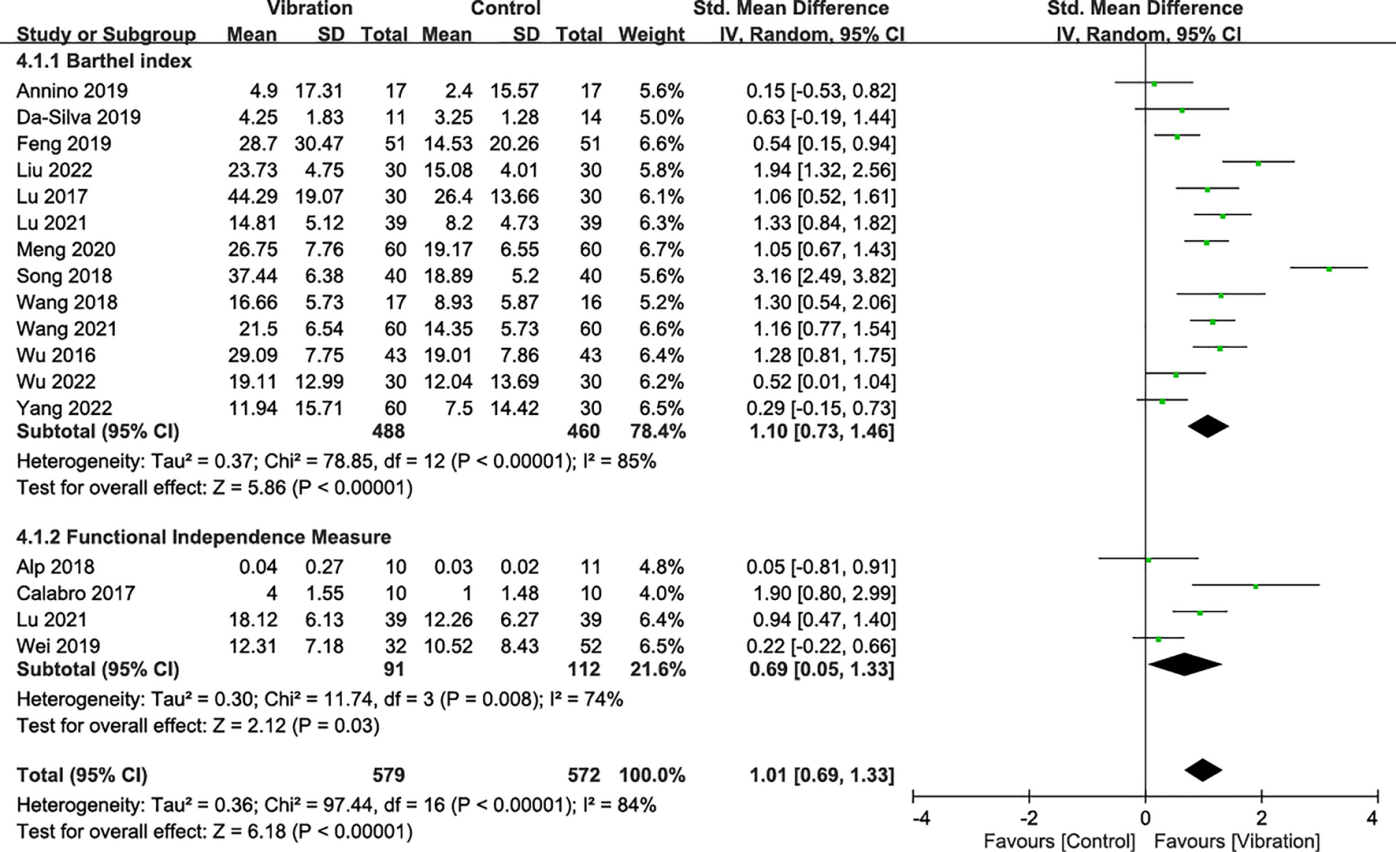
Forest plot showing the effects of vibration therapy on disability

A total of 16 RCTs reported disability outcomes that were assessed by the Functional Independence Measure (4 RCTs [10, 37, 47, 56]) and Barthel index (13 RCTs [38, 42, 43, 45–48, 51, 54, 55, 57–59]). Meta-analysis results showed that VT had favorable effects on score changes regarding the Barthel index (SMD = 1.10; *p* < 0.00001) and the Functional Independence Measure (SMD = 0.69; *p* = 0.03) compared with the control groups (Fig. 4). The combined results showed that VT achieved significantly greater changes in disability indices with an SMD of 1.01 (95% CI 0.69–1.33; *p* < 0.00001;* I*^2^: 84%) than the controls, regardless of the follow-up duration, intervention design, and type of vibration (Fig. 4).

Subgroup analysis results indicated significant differences between the treatment designs (*I*^2^: 82.6%; *p* = 0.02) and vibration types (*I*^2^: 84.7%; *p* = 0.01; Supplementary Table 4). People with stroke appeared to obtain favorable effects (SMD = 1.03) in response to VT combined with SR compared with those who received VT alone (SMD = 0.21). In addition, FMV (SMD = 2.14) appeared to achieve better treatment effects than WBV (SMD = 0.75). For disability, interventions lasting 4–8 weeks (SMD = 0.84) and 8 weeks or more (SMD = 0.87) yielded better results (Supplementary Table 4).

### Side effects and compliance

No clinically relevant adverse events, side effects (e.g., pain), or serious complications were reported after VT interventions in the included RCTs. Furthermore, good tolerance and compliance for FMV therapy was reported by three RCTs [42, 50, 52].

### Publication bias

The funnel plots of the effect sizes of each primary outcome measure are presented in Supplementary Fig. 1. No substantial asymmetry was identified by visual inspection of the funnel plots for UE motor impairment, UE motor function, and disability. The Egger’s linear regression test for each main outcome did not indicate any evidence of obvious reporting bias among the comparisons (All *p* > 0.05).

## Discussion

Regardless of whether people with stroke are in the acute, subacute, or chronic stage, FMV and WBV are effective for improving upper extremity motor impairment and enhancing motor function. Local vibration acts as a source of proprioceptive stimuli, inducing somatosensory and sensorimotor improvements through cortical reorganization, although disability benefits are only seen in the acute and subacute stages. VT can effectively improve upper limb motor impairments and function in people with chronic stroke. However, to enhance their independence in daily activities, a more comprehensive approach may be needed, potentially including improvements in environmental adaptation and cognitive skills and addressing psychosocial factors. Neuroplasticity significantly declines in the chronic stage after stroke, which necessitates the use of multiple therapies to promote overall recovery. In this regard, adjunct therapy combined with VT is more effective than monotherapy, particularly in improving functional impairments in the chronic stage, and could help enhance recovery outcomes in poeple with stroke.

### Disease stage, treatment design, and vibration type

In the disease stage, whether the person is in the acute, subacute, or chronic stage, using FMV or WBV is effective for improving motor impairment and enhancing motor function, with no difference between the two treatments. VT could be regarded as proprioceptive training that can induce meaningful somatosensory and sensorimotor functional improvements by means of cortical reorganization [62]. However, in terms of disability, benefits are only noticeable in the acute and subacute stages.

The tools that are used to assess disability status (such as the Functional Independence Measure or Barthel Index) focus on overall functionality [25, 26], rather than on a single motor function. Previous studies have suggested that specific training can significantly improve motor function in people with stroke but these improvements do not necessarily translate directly into independence in activities of daily living [63, 64]. In recent years, some studies have started to focus on reablement, a rehabilitation approach conducted in the individuals' living environment to help them relearn and regain essential life skills [65, 66]. VT may effectively improve motor impairments and enhance motor function in people with chronic stroke, but improving their independence in daily activities may require a more comprehensive approach. In addition to rehabilitation, enhancing environmental adaptation and cognitive abilities and addressing psychosocial factors are crucial considerations.

The treatment design of VT shows that adjunct therapy benefits are superior to those of monotherapy. Previous systematic reviews have also suggested that adjunct therapy is more effective than monotherapy in people with stroke. Neuroplasticity is higher in the early rehabilitation stage after a stroke, but in the chronic stage, the adaptability and self-repair capacity of the nervous system decrease significantly [67, 68]. Different therapies may have synergistic effects, enhancing overall recovery by addressing different mechanisms of stroke recovery [69]. Particularly for improving disability in the chronic stage, vibration therapy should be considered as part of adjunct therapy.

### Vibration frequency, intervention duration, and follow-up duration

Although both low (≤ 20 Hz) and high (> 20 Hz) frequencies can improve motor impairment and enhance motor function, as well as disability, with no significant difference between the two strategies, it appears that low frequency offers better benefits. Previous studies have indicated that VT can stimulate the excitation of the neuromuscular system and muscle strength [8]. Vibration can enhance the function of the spinal cord and cerebral cortex by stimulating Ia afferent signals from muscle spindles [10, 40]. Previous studies have also suggested that low-frequency vibration interventions can trigger somatosensory feedback to improve limb function and alleviate spasticity symptoms in people with stroke [16, 70].

Intervention times of 4–8 weeks and longer than 8 weeks are both effective for motor impairment, enhancing motor function, and disability, but intervention times of less than 4 weeks are only helpful for improving motor impairment. Previous meta-analyses on vibration also suggest that short-term vibration interventions are not helpful for reducing spasticity in people with stroke, with the effect size increasing gradually with the duration of vibration [18]. The effectiveness of training is closely related to the length of the training time; sufficient training time is necessary to achieve significant therapeutic effects [71]. Short VT intervention times may not provide adequate stimulation, thus limiting the effectiveness of the treatment.

Vibration intervention has significant effects in the short term (< 1 month and 1–3 months), but no sustained benefits were observed during the follow-up period of ≥ 3 months. Previous studies have indicated that insufficient training makes limb paralysis one of the major challenges faced by people with stroke after discharge [56]. People with stroke who frequently use the affected arm after discharge tend to experience better recovery in arm function [56]. Previous meta-analyses have indicated that high-intensity training post-stroke is crucial, and the more intense the training, the better the effect. An important principle in motor learning is repeated practice; when neurons are activated for a long duration simultaneously, the connections among them become stronger [72]. This suggests that in people with stroke, maintaining therapeutic effects in the long term may require continuous training interventions.

### Side effects and compliance

In this meta-analysis, no clinically relevant adverse effects were reported in the collected studies and the training protocols were well tolerated in two studies [50, 53]. The impact induced by high dosage vibration was reported to increase the risk of deteriorating osteoporosis, lower back pain, and complementary disability [73]; thus, the target location and direction of therapeutic equipment are crucial to prevent damage. Moreover, dizziness or muscle soreness were noted in 2.4–3.6% of people with stroke undergoing VT with stroke and cerebral palsy, respectively [74, 75]. WBV was reported to induce warm feet, dizziness, severe hip discomfort, and jaw or neck pain due to vibration [6]. A frequency higher than 20 Hz accompanied with a greater G value caused resonance, trauma, and dizziness, whereas that higher than 30 Hz led to discomfort and damage of fragile bones [74, 76].

### Limitations

Our study had several limitations. First, function and disability are interrelated, and while clinical assessment tools have primary measurement goals, they cannot solely assess motor function or disability elements alone. The mixed type of outcome results may have been a confounding factor. Second, more than two motor function outcome measures were used in some studies, which may have affected the results of the analysis. To maintain low heterogeneity, the Fugl-Meyer Assessment scale results were considered important and selected for the analysis.

## Conclusions

Our meta-analysis indicates that VT for improving upper limb function and disability recovery, especially when combined with SR, is both a reliable and safe therapeutic method. Initiating VT as early as possible post-stroke and a minimum of 4–8 weeks of VT is necessary to achieve improvements in upper extremity motor function after stroke. Both low and high vibration frequencies are effective, with FMV showing superior results compared with WBV, particularly for disability recovery. The effectiveness of VT is also influenced by factors such as the type of vibration, intervention mode, and follow-up duration. Maintaining the therapeutic effects of vibration therapy in the long term may require continuous interventions for people with stroke.

## Supplementary Information

Additional file 1.

Additional file 2.

Additional file 3.

Additional file 4.

Additional file 5.

## Data Availability

No datasets were generated or analysed during the current study.

## Abbreviations

CI: Confidence interval
CIs: Confidence intervals
FMV: Focal muscle vibration
MQ: Methodological quality
PEDro: Physiotherapy evidence database
RCTs: Randomized controlled trials
SD: Standard deviation
SMD: Standard mean difference
SMDs: Standard mean differences
SR: Standard rehabilitation
UE: Upper extremity
UEs: Upper extremities
VT: Vibration therapy
WBV: Whole-body vibration
