# Supplementary figures and images for: Upper extremity function and disability recovery with vibration therapy after stroke: a systematic review and meta-analysis of RCTs
Source: J Neuroeng Rehabil. 2024 Dec 21;21:221. doi: 10.1186/s12984-024-01515-6 (PMC11662454; doi:10.1186/s12984-024-01515-6)

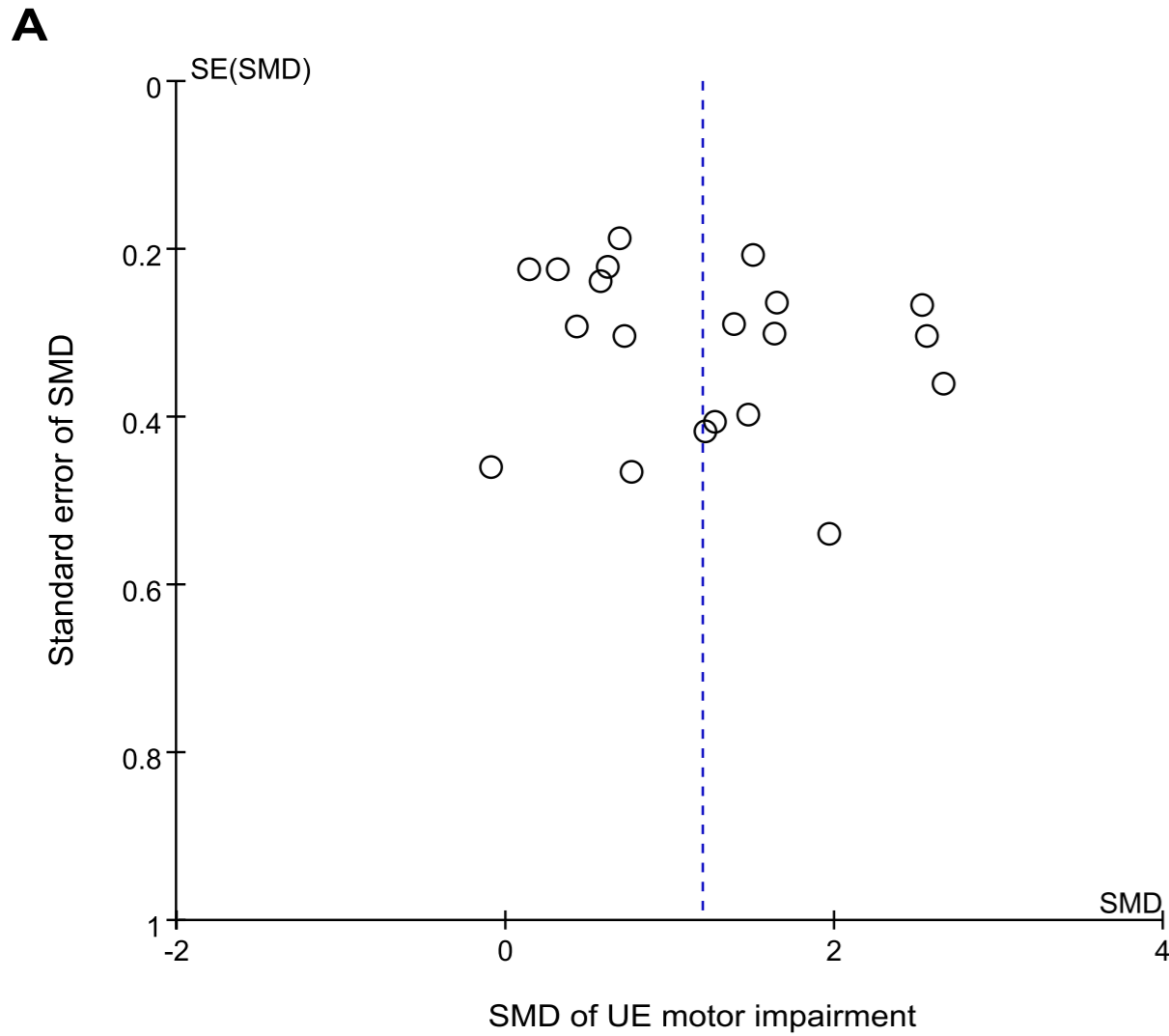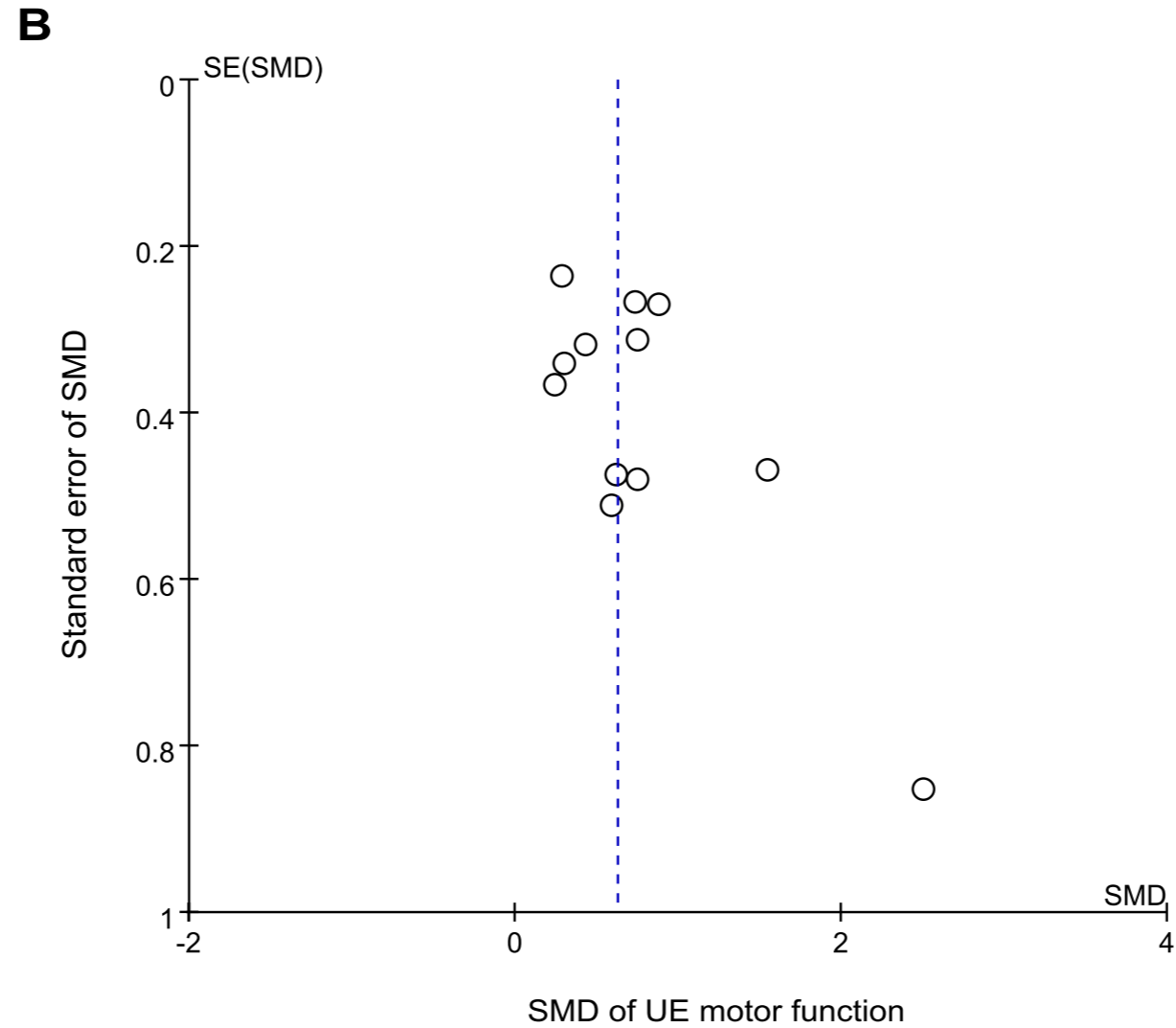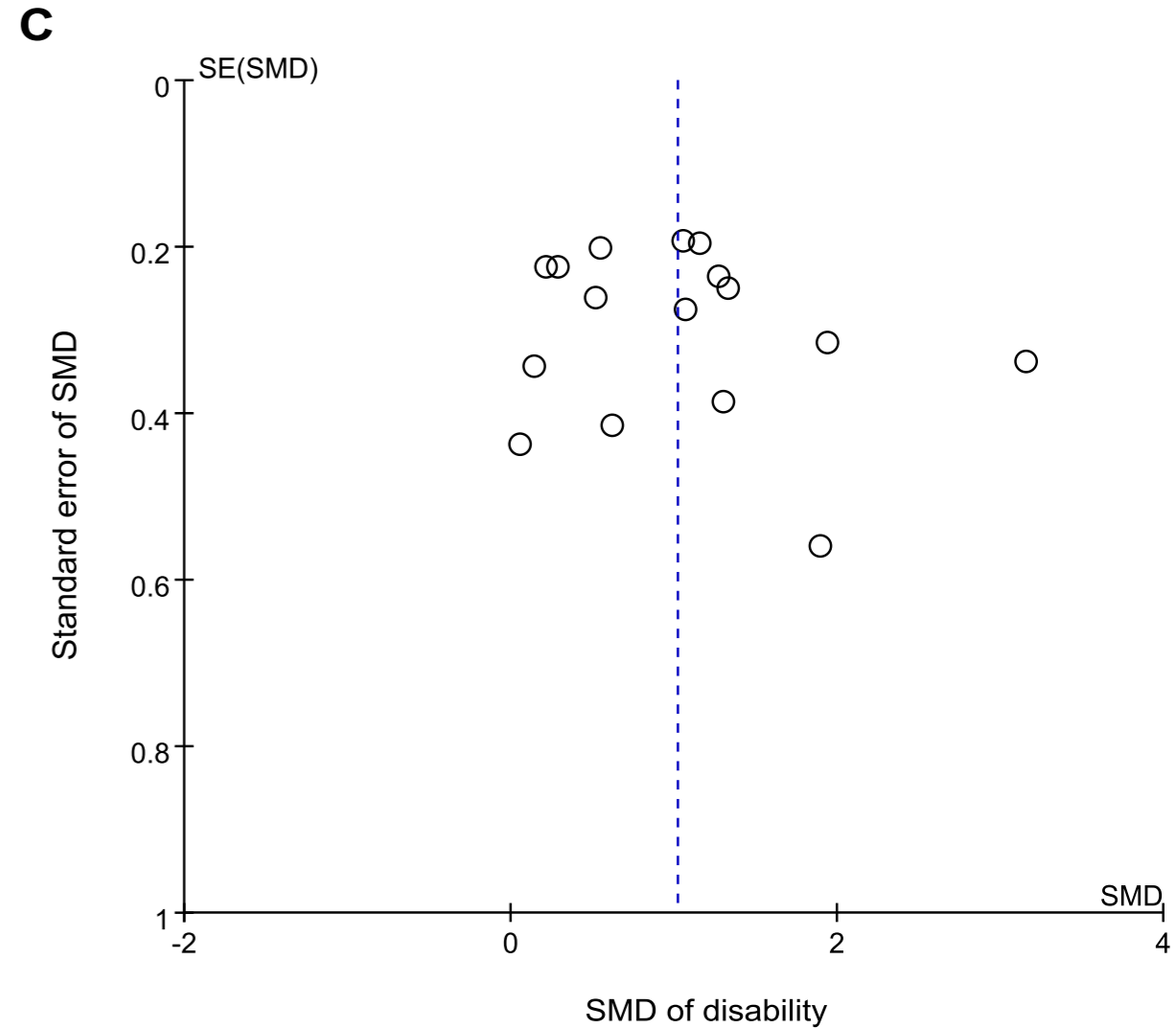

Supplement: Supplementary file 1 — Additional file 1. [file 12984_2024_1515_MOESM1_ESM.pdf]
