# Supplementary material for: Upper extremity function and disability recovery with vibration therapy after stroke: a systematic review and meta-analysis of RCTs
Source: J Neuroeng Rehabil. 2024 Dec 21;21:221. doi: 10.1186/s12984-024-01515-6 (PMC11662454; doi:10.1186/s12984-024-01515-6)
Supplement: Supplementary file 2 — Additional file 2. [file 12984_2024_1515_MOESM2_ESM.pdf]

Supplementary Table 1

Database search formulas

Data base	Search terms for query
**Pubmed**	
#1	(((stroke) OR cerebral vascular accident) OR hemiplegia) OR hemiparesis
#2	vibration
#3	(upper extremity) OR upper limb
#4	(Randomized controlled trial) OR Randomization
#5	(((#1) AND #2) AND #3) AND #4
**Physiotherapy Evidence Database (PEDro)**	
	Method: clinical trial
	Abstract & Title:
#1	stroke
#2	cerebral vascular accident
#3	vibration
**Excerpta Medica dataBASE (EMBASE)**	
#1	stroke
#2	cerebral vascular accident
#3	#1 OR #2
#4	vibration
#5	#3 AND #4 AND [randomized controlled trial]/lim) AND [humans]/lim (Randomized controlled trial) OR Randomization
**Cochrane Library Database**	
#1	stroke
#2	vibration
#3	randomized controlled trial
#4	#1 AND #2 AND #3
**China knowledge resource integrated database**	
#1	stroke
#2	vibration
#3	randomized controlled trial
#4	#1 AND #2 AND #3

(continued)

Table S1. (continued)

Data base	Search terms for query
**Google Scholar**	
#1	allintitle: stroke
#2	allintitle: vibration
#3	allintitle: randomized controlled trial
