# Supplementary material for: Upper extremity function and disability recovery with vibration therapy after stroke: a systematic review and meta-analysis of RCTs
Source: J Neuroeng Rehabil. 2024 Dec 21;21:221. doi: 10.1186/s12984-024-01515-6 (PMC11662454; doi:10.1186/s12984-024-01515-6)
Supplement: Supplementary file 3 — Additional file 3. [file 12984_2024_1515_MOESM3_ESM.pdf]

Supplementary Table 2

Type of vibration characteristics and application parameters

				Vibration treatment protocol for experimental group									Control group
							Application parameters (per session)						
Study Author (year)	Vibration type	Device	Manufacture (country)	Treatment posture	Targeted muscle	Time of application	Rate (Hz per sec)	Amplitude (mm)	Time (min/course x course/session)	Treatment frequency (session/week)	Treatment duration (week)	Number of treatment sessions	
Ahn (2019)	WBV	Galileo 2000	Novotec Medical GmbH (Germany)	Sit	Bilateral arms	Prior to SR	4–11	NR	30 x 1	5	4	20	No vibration
Alp (2016)	WBV	Compex Winplate	Chattanooga (USA)	Stand	Bilateral arms (with hands holding on the platform)	Following SR	40	4	5 x 1	3	4	12	Sham vibration (no stimuli)
Lee (2016)	WBV	Galileo tilting table system (vertical sinusoidal vibration)	Novotec Medical GmbH (Germany)	Sit	Bilateral arms (with hands holding on the platform)	Following SR	5–15	1–6	30 x 1	3	4	12	No vibration
Li (2020)	WBV	Galileo Med M Plus	Novotec Medical GmbH (Germany)	Sit	Bilateral arms (with hands holding on the platform)	NR	5–20	4	30 x 1	5	3	15	No vibration
Lu (2017)	WBV	BodyGreen	BODYGREEN (Taiwan)	Stand	Bilateral arms (with hands holding on the platform)	Following SR	20	4	20 x 1	5	8	40	No vibration
Wang (2018)	WBV	Wellengang Schwingungstrainer	SVG Medizinsysteme GmbH & Co. KG (Germany)	Sit	Involved arm (with paretic hand holding on the platform)	Following SR	5±1	4	10 x 1	6	4	24	No vibration
Wang (2021)	WBV	WBV advice	Shandong Zepu Medical Technology Co., Ltd (China)	Stand	Bilateral hands held arms of the device.	NR	3–50	6	20 x 1	5	4	20	No vibration
Wu (2022)	WBV	Galileo Med M Plus	Novotec Medical GmbH (Germany)	Stand	Bilateral arms (with hands holding on the platform)	Following SR	5–20	4	5 x 4	5	6	30	No vibration
Yang (2022)	WBV	Wellengang	SVG (Germany)	Stand Sit	Bilateral arms (with hands holding on the platform)	Following SR	30	4	5 x 4	6	8	48	No vibration

Annino (2019)	FMV	NR	NR	NR	Triceps	Following SR	30	2	5 x 1	3	8	24	No vibration
Calabrò (2017)	FMV	Pneumatic vibrator	NR	Sit	Triceps, supraspinatus, deltoid	Simultaneously applied with SR	80	0.3±0.1	60 x 1	5	8	40	Sham vibration (80 Hz, 0.1 mm)
Caliandro (2012)	FMV	Electromagnetic vibrator	NR	Supine		Prior to SR	100	0.2–0.5	10 x 3	3	1	3	Sham vibration (no stimuli)
Casale (2014)	FMV	VI-BRA, @Circle		NR	Triceps	Following SR	100	2 (2 cm^2, 250 mBar)	30 x 1	5	2	10	Sham vibration (no stimuli)
Celletti (2017)	FMV	Electromagnetic vibrator	NR	Supine	Pectoralis minor, biceps, flexor carpi muscle	NR	100	0.2–0.5	10 x 3	3	1	3	No vibration
Cordo (2022)	FMV	Electromagnetic vibrator	NR	Sit	wrist, hand	NR	60-80	0.2–0.3	5 x 6	2-3	6-10	18	No vibration
Da-Silva (2019)	FMV	CueS wristband (Axivity WAX9 tri-axial accelerometer)	Axivity, Newcastle upon Tyne (UK)	NA	Involved arm (wrist muscles)	Following SR	100	Dynamic range: ±8 g	12 hours/day	7	4	28	Sham vibration (no stimuli)
Feng (2019)	FMV	EV2610	Panasonic (Japan)	Supine	Upper arm (biceps), forearm	Following SR	25	NR	10 x 1	5	4	20	No vibration
Hsu (2021)	FMV	Perturbation-based pinch device (horizontal vibration produced by recoil-type actuators)	NR	Sit	Hand, pinch training (index finger and thumb)	Following SR	30	2	20 x 1	2	6	12	No vibration
Liu (2022)	FMV	Wellengang	SVG (Germany)	Sit	Shoulder muscle, biceps, wrist extensor, palm muscle	Following SR	6-20	4	3 x 4	5	4	20	Sham vibration
Lu (2021)	FMV	DMS100	QISHENG (China)	Supine	Shoulder muscle, biceps, wrist extensor, palm muscle	Following SR	60	6	3–6 x 2	5	4	20	No vibration
Meng (2020)	FMV	Galileo Med M Plus	Novotec Medical GmbH (Germany)	Sit	Bilateral arms (with hands holding on the platform)	NR	10	4	20 x 1	3	4	12	No vibration
Oliveira (2018)	FMV	Digital Vibration Pad	NissanFisio (Brazil)	Sit	Involved UL	NA (no SR)	35	1.5	15 x 1	3	4	12	No vibration

Seo (2019)	FMV	C-3 Tactor	Engineering Acoustics Inc. (USA)	Sit	Wrist muscle	Simultaneously applied with SR	500	60% of the sensory threshold	15 x 1	3	2	6	Sham vibration (no stimuli)
Song (2018)	FMV	ZEPU-K5000A	Shandong Zepu Medical Technology Co., Ltd (China)	Sit or supine	Upper arm, forearm, wrist, hand	Following SR	15–60	6	20 x 1	5	3	15	No vibration
Tavernese (2013)	FMV	Horus (acoustic-wave vibratory energy)	Akropolis (Italy)	Supine	Biceps brachii, flexor carpii ulnaris	Following SR	120	10	30 x 1	5	2	10	No vibration
Toscano (2019)	FMV	Cro®System (electromechanical transducer)	NEMOCO srl (Italy)	Supine	Biceps brachii, flexor carpii radialis	NR	100	0.2–0.5	10 x 3	3	1	3	Sham vibration (no stimuli)
Wei (2019)	FMV	Sensory Cueing Wristwatch (SCW-V2)	PolyU Technology and Consultancy Co., Ltd. (China)	NA	Involved arm (wrist muscles)	Simultaneously applied with SR	196	NR	3 hours/day	7	4	28	Sham vibration (no stimuli)
Wu (2016)	FMV	EV2610	Panasonic (Japan)	Supine	Upper arm (biceps), forearm	Following SR	25	NR	10 x 1	5	8	40	No vibration
Yuan (2018)	FMV	Deep muscle stimulator	DMS (Japan)	NR	Shoulder muscle, biceps, wrist extensor, palm muscle	NR	60	6	4–8 x 3	5	4	20	Sham vibration (no contact)
Zhu (2017)	FMV	Dynamic monitor system	DMS (USA)	Sit or supine	Upper arm, forearm, wrist, hand	NR	60	6	20 x 1	7	3	21	No vibration

[a]Use of a regional block or anesthesia was allowed. No patient received supplementation of local anesthesia in the area of treatment.

EMS = Electro Medical Systems; HMT = High Medical Technology; EFD = energy flux density; TED = Total energy dose (intensity × number of shocked impulses); NR = not Reported; NA, not application; UE, upper limb.
