# Supplementary material for: Upper extremity function and disability recovery with vibration therapy after stroke: a systematic review and meta-analysis of RCTs
Source: J Neuroeng Rehabil. 2024 Dec 21;21:221. doi: 10.1186/s12984-024-01515-6 (PMC11662454; doi:10.1186/s12984-024-01515-6)
Supplement: Supplementary file 4 — Additional file 4. [file 12984_2024_1515_MOESM4_ESM.docx]

**Supplementary Table 3**

| Summary of the methodological quality of the included studies^a^ | | | | | | | | | | | | | |
| --- | --- | --- | --- | --- | --- | --- | --- | --- | --- | --- | --- | --- | --- |
| Study, first author year | Overall^b^ | Eligibility criteria^c^ | 1 | 2 | 3 | 4 | 5 | 6 | 7 | 8 | 9 | 10 |  |
| Ahn 2019 | 6/10^d^ | *X* | *X* |  | *X* |  |  |  | *X* | *X* | *X* | *X* |  |
| Alp 2018 | 7/10^d^ | *X* | *X* |  | *X* | *X* |  | *X* | *X* |  | *X* | *X* |  |
| Annino 2019 | 5/10 | *X* | *X* |  | *X* |  |  | *X* | *X* |  |  | *X* |  |
| Calabro 2017 | 7/10 | *X* | *X* |  | *X* | *X* |  | *X* | *X* |  | *X* | *X* |  |
| Caliandro 2012 | 9/10^d^ | *X* | *X* | *X* | *X* | *X* |  | *X* | *X* | *X* | *X* | *X* |  |
| Casale 2014 | 7/10 | *X* | *X* |  | *X* | *X* |  | *X* | *X* |  | *X* | *X* |  |
| Celletti 2017 | 8/10 | *X* | *X* | *X* | *X* |  |  | *X* | *X* | *X* | *X* | *X* |  |
| Cordo 2022 | 7/10 | *X* | *X* |  | *X* | *X* |  | *X* | *X* |  | *X* | *X* |  |
| Da-Silva 2019 | 5/10^d^ | *X* | *X* |  | *X* | *X* |  | *X* |  |  |  | *X* |  |
| Feng 2019 | 6/10 | *X* | *X* |  | *X* |  |  |  | *X* | *X* | *X* | *X* |  |
| Hsu 2021 | 7/10 | *X* | *X* | *X* | *X* |  |  | *X* | *X* |  | *X* | *X* |  |
| Lee 2016 | 7/10 | *X* | *X* |  | *X* |  |  | *X* | *X* | *X* | *X* | *X* |  |
| Li 2020 | 6/10 | *X* | *X* |  | *X* |  |  |  | *X* | *X* | *X* | *X* |  |
| Liu 2022 | 7/10 | *X* | *X* |  | *X* | *X* |  |  | *X* | *X* | *X* | *X* |  |
| Lu 2017 | 6/10 | *X* | *X* |  | *X* |  |  |  | *X* | *X* | *X* | *X* |  |
| Lu 2021 | 6/10 | *X* | *X* |  | *X* |  |  |  | *X* | *X* | *X* | *X* |  |
| Meng 2020 | 6/10 | *X* | *X* |  | *X* |  |  |  | *X* | *X* | *X* | *X* |  |
| Oliveira 2018 | 6/10^d^ | *X* | *X* |  | *X* |  |  |  | *X* | *X* | *X* | *X* |  |
| Seo 2019 | 9/10 | *X* | *X* |  | *X* | *X* | *X* | *X* | *X* | *X* | *X* | *X* |  |
| Song 2018 | 6/10 | *X* | *X* |  | *X* |  |  |  | *X* | *X* | *X* | *X* |  |
| Tavernese | 8/10 | *X* | *X* | *X* | *X* |  |  | *X* | *X* | *X* | *X* | *X* |  |
| Toscano 2019 | 9/10 | *X* | *X* |  | *X* | *X* | *X* | *X* | *X* | *X* | *X* | *X* |  |
| Wang 2018 | 6/10 | *X* | *X* |  | *X* |  |  |  | *X* | *X* | *X* | *X* |  |
| Wang 2021 | 6/10 | *X* | *X* |  | *X* |  |  |  | *X* | *X* | *X* | *X* |  |
| Wei 2019 | 8/10 | *X* | *X* | *X* | *X* | *X* |  | *X* |  | *X* | *X* | *X* |  |
| Wu 2016 | 6/10 | *X* | *X* |  | *X* |  |  |  | *X* | *X* | *X* | *X* |  |
| Wu 2022 | 6/10 | *X* | *X* |  | *X* |  |  |  | *X* | *X* | *X* | *X* |  |
| Yang 2022 | 6/10 | *X* | *X* |  | *X* |  |  |  | *X* | *X* | *X* | *X* |  |
| Yuan 2018 | 7/10 | *X* | *X* |  | *X* | *X* |  |  | *X* | *X* | *X* | *X* |  |
| Zhu 2017 | 6/10 | *X* | *X* | *X* | *X* |  |  |  | *X* |  | *X* | *X* |  |
| Summary^e^ |  | 30 | 30 | 6 | 30 | 11 | 2 | 14 | 28 | 23 | 28 | 30 |  |
| ^a^PEDro, Physiotherapy Evidence Database. The guideline for the PEDro scale is available from the PEDro database (<https://www.pedro.org.au/english/downloads/pedro-scale/>).  ^b^Points of methodological quality are denoted as “*X*” for fulfilled criteria.  ^c^This item is not used to calculate the total score.  ^d^The score was determined by a third assessor.  ^e^This was calculated as the number of studies satisfied.  PEDro classification scale: 1 = random allocation, 2 = concealed allocation, 3 = similarity at the baseline, 4 = subject blinding, 5 = therapist blinding, 6 = assessor blinding, 7 = >85% follow-up for at least one key outcome, 8 = intention-to-treat analysis, 9 = between-group statistical comparison for at least one key outcome, 10 = point and variability measures for at least one key outcome. Methodological quality: high, ≥7 points; medium, 4–6 points; low, ≤3 points. | | | | | | | | | | | | | |
