# Supplementary material for: Upper extremity function and disability recovery with vibration therapy after stroke: a systematic review and meta-analysis of RCTs
Source: J Neuroeng Rehabil. 2024 Dec 21;21:221. doi: 10.1186/s12984-024-01515-6 (PMC11662454; doi:10.1186/s12984-024-01515-6)
Supplement: Supplementary file 5 — Additional file 5. [file 12984_2024_1515_MOESM5_ESM.docx]

**Supplementary Table 4**

| Summary of the subgroup analyses results of the treatment effects on the main outcomes | | | | | | | | | | | | | | | | | | |
| --- | --- | --- | --- | --- | --- | --- | --- | --- | --- | --- | --- | --- | --- | --- | --- | --- | --- | --- |
| Subgroup | Upper-extremity motor impairment | | | | |  | Upper-extremity motor function | | | |  |  | Disability | | | | | |
|  | Comparison, *n* | SMD | (95% CI) | *P*-value | *I*^2^ (%) |  | Comparison, *n* | SMD | (95% CI) | *P*-value | *I*^2^ (%) |  | Comparison, *n* | SMD | (95% CI) | *P*-value | *I*^2^ (%) |  |
| MQ level |  |  |  |  |  |  |  |  |  |  |  |  |  |  |  |  |  |  |
| PEDro score <7/10 | 12 | 1.43 | (0.96, 1.89)^‡^ | <0.00001 | 89 |  | 5 | 0.85 | (0.54, 2.47)^†^ | <0.00001 | 0 |  | 12 | 0.99 | (0.63, 1.36)^‡^ | <0.00001 | 84 |  |
| PEDro score ≥7/10 | 8 | 0.80 | (0.38, 1.21)^‡^ | 0.0002 | 69 |  | 7 | 0.47 | (0.22, 0.73)^†^ | 0.0003 | 26 |  | 4 | 1.00 | (-0.04, 2.03)^‡^ | n.s. | 89 |  |
| Subgroup difference |  |  |  | n.s. | 74.2 |  |  |  |  | n.s. | 59 |  |  |  |  | n.s. | 0 |  |
| Disease stage  (onset duration of stroke) |  |  |  |  |  |  |  |  |  |  |  |  |  |  |  |  |  |  |
| Acute (<3 months) | 11 | 1.35 | (0.88, 1.83)^‡^ | <0.00001 | 87 |  | 3 | 0.81 | (0.17, 1.45)^‡^ | 0.01 | 70 |  | 9 | 0.92 | (0.60, 1.24)^†^ | <0.00001 | 71 |  |
| Subacute (3−6 months) | 5 | 1.25 | (0.42, 2.08)^‡^ | 0.003 | 92 |  | 1 | 0.73 | (0.21,1.257) | 0.006 | NA |  | 5 | 1.13 | (0.22, 2.04)^‡^ | 0.01 | 93 |  |
| Chronic (>6 months) | 4 | 0.64 | (0.30, 0.98)^†^ | 0.0003 | 40 |  | 8 | 0.56 | (0.28, 0.84)^†^ | <0.0001 | 4 |  | 2 | 0.97 | (-0.74, 2.68)^‡^ | n.s. | 86 |  |
| Subgroup difference |  |  |  | n.s. | 56 |  |  |  |  | n.s. | 0 |  |  |  |  | n.s. | 0 |  |
| Treatment design |  |  |  |  |  |  |  |  |  |  |  |  |  |  |  |  |  |  |
| Monotherapy | 3 | 0.26 | (-0.13, 0.65)^†^ | n.s. | 0 |  | 5 | 0.68 | (0.34, 1.02)^†^ | <0.0001 | 49 |  | 2 | 0.21 | (-0.22, 0.64)^†^ | n.s. | 28 |  |
| Adjunct therapy | 17 | 1.18 | (0.81, 1.55)^‡^ | <0.00001 | 85 |  | 7 | 0.61 | (0.31, 0.92)^†^ | <0.0001 | 30 |  | 14 | 1.03 | (0.68, 1.37)^‡^ | <0.00001 | 84 |  |
| Subgroup difference |  |  |  | 0.0009 | 91 |  |  |  |  | n.s. | 0 |  |  |  |  | 0.02 | 82.6 |  |
| Vibration type |  |  |  |  |  |  |  |  |  |  |  |  |  |  |  |  |  |  |
| FMV | 13 | 1.07 | (0.62, 1.52)^‡^ | <0.00001 | 88 |  | 10 | 0.69 | (0.40, 0.98)^†^ | <0.00001 | 35 |  | 10 | 2.14 | (1.15, 3.13)^‡^ | <0.0001 | 96 |  |
| WBV | 7 | 1.39 | (0.84, 1.95)^‡^ | <0.00001 | 84 |  | 2 | 0.61 | (0.21, 1.01)^†^ | 0.003 | 0 |  | 6 | 0.75 | (0.36, 1.14)^‡^ | 0.0001 | 67 |  |
| Subgroup difference |  |  |  | n.s. | 0 |  |  |  |  | n.s. | 0 |  |  |  |  | 0.01 | 84.7 |  |
| Vibration frequency | |  |  |  |  |  |  |  |  |  |  |  |  |  |  |  |  |  |
| Low (≤20 Hz) | 7 | 1.46 | (0.96, 1.95)^‡^ | <0.00001 | 77 |  | 2 | 0.61 | (0.21, 1.01)^†^ | 0.003 | 0 |  | 5 | 1.15 | (0.72, 1.57)^‡^ | <0.00001 | 67 |  |
| High (>20 Hz) | 13 | 1.05 | (0.58, 1.52)^‡^ | <0.0001 | 89 |  | 10 | 0.69 | (0.40, 0.98)^†^ | <0.00001 | 35 |  | 10 | 0.91 | (0.46, 1.37)^‡^ | <0.0001 | 87 |  |
| Subgroup difference |  |  |  | n.s. | 26.7 |  |  |  |  | n.s. | 0 |  |  |  |  | n.s. | 0 |  |
| Intervention duration (week) |  |  |  |  |  |  |  |  |  |  |  |  |  |  |  |  |  |  |
| <4 | 5 | 1.51 | (0.66, 2.36)^‡^ | 0.0005 | 85 |  | 5 | 0.55 | (-0.03, 1.13)^‡^ | n.s. | 61 |  | 3 | 1.29 | (-0.74, 3.32)^‡^ | n.s. | 95 |  |
| 4−8 | 12 | 1.19 | (0.72, 1.66)^‡^ | <0.00001 | 89 |  | 8 | 0.57 | (0.31, 0.83)^†^ | <0.0001 | 6 |  | 10 | 0.84 | (0.53, 1.15)^‡^ | <0.00001 | 72 |  |
| ≥8 | 4 | 0.89 | (0.39, 1.39)^‡^ | 0.0005 | 68 |  |  |  |  |  |  |  | 5 | 0.87 | (0.31, 1.42)^‡^ | 0.002 | 77 |  |
| Subgroup difference |  |  |  | n.s. | 0 |  |  |  |  | n.s. | 0 |  |  |  |  | n.s. | 0 |  |
| Follow-up duration |  |  |  |  |  |  |  |  |  |  |  |  |  |  |  |  |  |  |
| <1 month | 5 | 1.51 | (0.66, 2.36)^‡^ | 0.0005 | 85 |  | 5 | 0.55 | (-0.03, 1.13)^‡^ | n.s. | 61 |  | 3 | 1.27 | (-0.79, 3.32)^‡^ | n.s. | 96 |  |
| ≥1 months, <3 months | 16 | 1.12 | (0.75, 1.49)^‡^ | <0.00001 | 87 |  | 9 | 0.07 | (0.48, 0.70)^†^ | <0.0001 | 4 |  | 16 | 0.66 | (0.26, 1.06)^‡^ | 0.001 | 88 |  |
| ≥3 months | 3 | 0.07 | (-0.29, 0.43)^†^ | n.s. | 0 |  | 1 | 0.76 | (-0.18, 1.70) | n.s. | NA |  | 3 | 0.48 | (-0.60, 1.55)^†^ | n.s. | 82 |  |
| Subgroup difference |  |  |  | <0.0001 | 90 |  |  |  |  | n.s. | 0 |  |  |  |  | n.s. | 0 |  |
| ^†^Fixed-model effect  ^‡^Random-model effect  FMV, focal muscle vibration; WBV, whole-body vibration; SMD, standard mean difference; *I*^2^, heterogeneity; MQ, methodological quality; PEDro, Physiotherapy Evidence Database; n.s., non-significant (*P*>0.05); NA, not applicable; CI, confidence interval. | | | | | | | | | | | | | | | | | | |
